# Production and characterization of a promising microbial-derived lipase enzyme targeting *BCL-2* gene expression in hepatocellular carcinoma

**DOI:** 10.1186/s12934-025-02671-7

**Published:** 2025-03-08

**Authors:** Amal M. Abo-Kamer, Ahmed A. Abdelaziz, Esraa S. Elkotb, Lamiaa A. Al-Madboly

**Affiliations:** Department of Microbiology and Immunology, Faculty of Pharmacy, Tanta University, Tanta, Egypt

**Keywords:** Optimization, Mutant lipolytic activity, Screening, Biological samples, Lipase, Anticancer, Hepatocellular carcinoma (HepG-2)

## Abstract

**Context and goal:**

This study aimed to isolate and optimize a high-yield lipase-producing *Pseudomonas aeruginosa* strain from biological samples, enhance enzyme production through random mutagenesis, and evaluate its potential anticancer activity. Fifty-one biological samples (blood, urine, sputum, wound pus) were screened, and three isolates demonstrated significant lipase activity. The isolate with the highest activity, identified as *P. aeruginosa* (GenBank accession number PP436388), was subjected to ethidium bromide-induced mutagenesis, resulting in a two-fold increase in lipase activity (312 U/ml). Lipase production was optimized using submerged fermentation, with critical factors identified statistically as Tween 80, peptone, and substrate concentration. The enzyme was purified via ammonium sulfate precipitation and Sephadex G-100 chromatography, and its molecular weight (53 kDa) was confirmed by SDS–PAGE.

**Findings:**

Optimal conditions for enzyme production included a pH of 9, temperature of 20 °C, and a 24-h incubation period. The partially purified enzyme exhibited high stability at pH values up to 10 and storage temperatures of 4 °C. Anticancer activity was evaluated using the MTT assay, revealing an IC_50_ of 78.21 U/ml against human hepatocellular carcinoma using HepG-2 cells, with no cytotoxicity observed against Vero cells. Flow cytometry confirmed that the enzyme’s anticancer potential was mediated through apoptosis and necrosis. QRT-PCR data revealed that the expression of the *Bcl-2* gene was significantly downregulated by 62% (*P* < 0.05) following the treatment of HepG-2 cells with the lipase enzyme. These findings suggest that lipase from *P. aeruginosa* holds promise as a novel therapeutic agent for hepatocellular carcinoma, addressing the limitations of current treatments.

## Introduction

Lipases, also known as triacylglycerol acyl hydrolases, are a class of hydrolytic enzymes that catalyze the breakdown of triglycerides into glycerol and free fatty acids. These enzymes have garnered significant attention for their versatility, stability, and applicability in various industries, including pharmaceuticals, food processing, and environmental biotechnology. Among microbial lipases, those derived from *Pseudomonas aeruginosa* stand out due to their remarkable catalytic efficiency, broad substrate specificity, and stability under harsh environmental conditions, making them highly suitable for both industrial and therapeutic applications [1].

To fully exploit the potential of *P. aeruginosa* lipase, optimizing its production is critical. Factors such as temperature, pH, carbon and nitrogen sources, and specific inducers significantly influence enzyme yield and activity. Advanced optimization strategies, including Response Surface Methodology (RSM) and Design of Experiments (DoE), have been employed to systematically enhance production by identifying optimal combinations of these parameters [2]. Once produced, the enzyme must be purified for downstream applications. Techniques such as ammonium sulfate precipitation, ion-exchange chromatography, and gel filtration chromatography are widely utilized to achieve high-purity preparations with enhanced stability and activity [3].

Recent research has unveiled promising therapeutic applications of *P. aeruginosa* lipase, particularly in oncology. Hepatocellular carcinoma (HCC), one of the most aggressive forms of liver cancer, poses a significant global health challenge due to its high mortality rate and limited treatment options. Lipases have demonstrated anticancer potential through mechanisms such as apoptosis induction, disruption of lipid metabolism critical for cancer cell survival, and generation of reactive oxygen species (ROS). Lipases can hydrolyze lipids, producing bioactive molecules like ceramides, which activate apoptosis pathways by affecting mitochondrial function and caspases. These enzymes can activate both intrinsic (mitochondrial) and extrinsic (death receptor) pathways, initiating apoptosis through caspase activation [4]. These findings suggest that microbial lipases could serve as innovative biotherapeutic agents in cancer treatment [5, 6]. This study provides a comprehensive examination of *P. aeruginosa* lipase, focusing on its production, optimization, purification, and emerging role in HCC therapy. By addressing existing challenges and exploring novel applications, this work aims to advance the understanding and utilization of this multifunctional enzyme in industrial and medical contexts.

## Materials and methods

### Media for isolation, screening, and optimization

These media are used for the isolation of *P. aeruginosa* from human specimens. To determine the best basal media for lipase production, three different media compositions have been chosen.

**Media No. 1 (M1)** composition (g/l).

Peptone from gelatin 17.0; peptone from casein 1.5; peptone from meat 1.5; sodium chloride 5.0; lactose 10.0; bile salt mixture 1.5; neutral red 0.03; crystal violet 0.001; agar–agar 13 [7].

**Media No. 2 (M2)** composition (g/l).

Gelatin peptone 20.0; magnesium chloride 1.4; potassium sulfate 10.0; cetrimide 0.3; glycerol 10.0; Agar 13.6 [8].

**Media No. 3 (M3)** composition (g/l).

Peptone 5.0; beef extract 3.0; calcium chloride 1 g/l; agar 15.0; phenol red dye 1 mg/ml [9].

#### Collecting samples and isolating bacteria

Fifty-one *P. aeruginosa* isolates were obtained from the laboratory of Tanta University Teaching Hospitals (Tanta, Gharbia Governorate, Egypt). They were recovered from different biological specimens including blood (*n* = 18), urine (*n* = 10), sputum (*n* = 11), and wound pus (*n* = 12) by the laboratory staff. The specimens were inoculated onto nutrient agar plates that were incubated at 37 °C for 24 h. To ensure pure cultures, isolated colonies were repeatedly picked and streaked on nutrient agar plates. The pure bacterial cultures were recultivated on cetrimide agar plates were stored in slants at a temperature of 4 °C for further analyses [1].

#### Primary screening for lipase producing bacteria

Isolates underwent screening for lipase production ability through the inoculation of bacterial colonies on primary phenol red agar media. The media composition included 5 g/l peptone, 3 g/l yeast extract, 15 g/l agar, 1 g/l CaCl_2_, and pH adjusted to 7.4 using 0.1 M NaOH in distilled water. The sterilization process involved autoclaving for 15 min at 121 °C with 15 lb pressure, followed by cooling to 60 °C. Subsequently, 10 ml/l phenol red dye (1 mg/ml) and 10 ml/l substrate (olive oil) were added. Aliquots were then transferred to Petri dishes and allowed to solidify. Each pure culture was streaked over phenol red agar with a loopful, and the mixture was cultured for 48 h at 37 °C.

The observation of a color change from pink to yellow indicated the release of fatty acids due to lipolysis. Lipase-positive strains were determined and recorded based on the yellow zone around bacterial colonies. The best lipase-producing bacteria out of 51 bacterial isolates was selected and sub-cultured on nutrient agar for further experimental studies. Each pure colony isolated and bacterial cells washed in a sterile saline (0.9% NaCl) before lipase assayed [1].

#### Secondary screening for lipase production

The cells of the selected lipase producing bacterial cultures were collected through centrifugation at a speed of 2800 × g for 15 min, washed with sterile saline then all were screened for production of lipase. The production media (pH 7.0) was prepared with 5 g/l peptone, 5 g/l beef extract in distilled water, autoclaved for 15 min at 15 lb pressure (121 °C) and cooled to about 60 °C before the addition of 10 ml/l olive oil. About 1 ml of overnight grown selected lipase producing bacterial cultures was inoculated in 100 ml of production medium in 250 ml Erlenmeyer flasks separately and incubated at 37 °C for 24 h. Culture was centrifuged at 10,000 rpm for 10 min after incubation, supernatant was used to assay for lipase [1].

#### Assay of lipase for selecting the most potent lipase-producing strain

To prepare the substrate emulsion, approximately 0.8 ml of olive oil was mixed with 99.2 ml of ethanol, and the mixture was stirred using a vortex to create a temporary stable and homogeneous emulsion which remains stable for a short period (minutes to an hour). This emulsion served as the substrate for the reaction. In the next step, the reaction mixture was prepared by adding 0.1 ml of the substrate emulsion (olive oil in ethanol) and 2 ml of 0.05 M phosphate buffer (pH 7.0) to a clean test tube or cuvette. The buffer maintained the optimal pH for enzyme activity. Then, 1 ml of crude lipase enzyme solution, which was the supernatant obtained from the secondary screening of lipase, was added to initiate the reaction. The mixture was gently vortexed to ensure uniform distribution of the enzyme and substrate.

Following this, the reaction mixture was incubated in a water bath at 37 °C for 5 min, with gentle mixing throughout the incubation period. After the 5-min incubation, the change in absorbance (ΔA) was measured using a spectrophotometer at 410 nm. This change in absorbance was due to the formation of free fatty acids, such as oleic acid from olive oil. The absorbance was recorded at the start and end of the incubation to calculate ΔA.

Finally, the lipase activity was calculated using the following equation:

$${\text{Lipase}}\,{\text{Activity}}({\text{U}}/{\text{mL}}) = (\Delta {\text{A}}/({\text{l}}*{\text{V}}\_{\text{s}}*{\text{t}}))*1953$$

where ΔA represents the change in absorbance at 410 nm, l is the path length of the cuvette (usually 1 cm), t is the reaction time (5 min in this case), V_s is the volume of enzyme used (in ml), and 1953 is the calibration factor specific to the assay for free fatty acid detection [10–12].

### Mutagenesis

The wild-type strain of *P. aeruginosa* Ps193 was cultivated in a nutrient broth medium for 2 days at a temperature of 28 ℃. Subsequently, 10 ml of the culture underwent centrifugation at a speed of 9000 g at a temperature of 4 ℃ for 10 min to separate the cell biomass. The resulting cell biomass pellet was then re-suspended in 10 ml of sterilized saline solution (0.9%). To induce mutagenesis using Ethidium bromide (Eth. Br), separate plates were treated with a concentration of 10 mg/ml Eth. Br and incubated at a temperature of 28 ℃ for 60 min. Following the mutagenesis treatments, the cells were collected through centrifugation at a speed of 2800 × g for 15 min, washed with sterile saline, the washed cells were transfered to nutrient agar plates and then incubated. The colonies are then screened for high efficiency lipase production using the method of lipase assay that mentioned before [13–15].

### Morphological, biochemical, and genetic identification of lipase-producing isolate

To obtain details on morphology, bacterial colonies were subjected to the standard Gram-stain and examined using an oil immersion high-power light microscopy (100 × magnification lens). Additionally, the colonies were cultured on cetrimide agar as a selective media. The bacterial isolate was subsequently analyzed using standard biochemical tests (such as citrate, catalase, and indole tests) for further identification [16].

#### 16S ribosomal RNA gene sequences identification and PCR amplification

Bacterial genomic DNA was extracted using the E.Z.N.A.^®^ Bacterial DNA Mini Kit (D3350-00S, OMEGA BIO-TEK, USA) following the manufacturer’s instructions. The DreamTaq Green PCR Master Mix (2 ×) (K1081, Thermo Fisher, USA) was utilized for the amplification of a specific gene as per the manufacturer’s protocol on the Creation (Holland, Inc.) Polymerase Chain Reaction (PCR) system cycler. A universal primer targeting the 16S rRNA gene was used as a molecular marker for the identification of the sample isolates. The following primer sequences were used for amplification of the 16S rRNA gene: forward primer 5′-AGAGTTTGAATCCTGGCTCAG-3′ and reverse primer 5′-AAGGAGGTGATCCAGCC-3′ [17]. The thermal cycling conditions included an initial denaturation at 95 °C for 5 min, followed by 35 cycles of denaturation at 95 °C for 1 min, annealing at 50 °C for 1.5 min, elongation at 72 °C for 1 min, and a final elongation at 72 °C for 10 min [17].

#### Data analysis

The Total Lab analysis program (www.totallab.com, Ver.1.0.1) was used to analyze data from the Gel documentation system (Geldoc-it, UVP, England). Positive amplicons measuring 1500 bp were extracted from the agarose gel. The resulting PCR products underwent purification using Micro spin filters and were quantified spectrophotometrically. Sequence analysis was conducted using the ABI PRISM^®^ 3100 Genetic Analyzer (Micron-Corp. Korea). The comparison between the generated and published sequences was determined by utilizing NCBI reference sequences with BLAST. To construct the phylogenetic tree, Clustal Omega software (https://www.ebi.ac.uk/Tools/msa/clustalo/) was employed [17].

### Enzyme production by submerged fermentation

The *P. aeruginosa* isolate with promising lipolytic activity was used to inoculate 50 ml of fermentation production media in a 250 ml Erlenmeyer flask. The basal media was adjusted to pH 7 before autoclaving using a pH meter from Japan. After 24 h of cultivation at 37 °C, the lipase activities were assessed. The composition of the basal production media (control) was as follows: Peptone 5.0 g/l, beef extract 1.0 g/l, yeast extract 2.0 g/l, sodium chloride 5.0 g/l, agar 15.0 g/l, and after cooling, 10 ml/l olive oil was added. The flasks were then incubated in a rotary shaker fermenter at 37 °C for 24 h and 150 rpm in a rotary shaker incubator (Stuart S1-500-UK). The bacterial suspensions were centrifuged at 10,000 rpm for 10 min, and the supernatant was collected and stored at 4 °C for further research as a crude extract for enzyme activity assay [11, 12, 18]. The primary production factors that may affect lipase production, such as sources of carbon and nitrogen, were considered during the enzyme assay performed according to standard procedures [19].

### Optimization of culture media conditions and medium components for best lipase production by using one-factor-at-a-time (OFAT) approach

#### Effect of various carbon sources on lipase production

Bacterial cells were cultivated on basal media (M3) with a variety of carbon sources including glucose, fructose, and tween 80 to induce lipase synthesis. Fermentation media with different carbon sources at concentrations of 5 g/l were inoculated and incubated at 37 °C for 24 h with an agitation speed of 150 rpm. This experiment was repeated three times. The culture filtrate obtained post-extraction was then employed for quantitative assessment of extracellular lipase. All other variables were kept constant at their optimal settings [18].

#### Effect of various nitrogen sources on lipase production

Various nitrogen sources, (such as peptone, yeast extract, and casein), were separately added to the fermentation broth media at a concentration of 5 g/l. The flasks containing the fermentation media were then incubated at 37 °C for 24 h with an agitation speed set at 150 rpm to investigate enzyme activity. This work was repeated three times. All other variables were maintained at their optimal levels [18].

### Multi-factorial experiments for optimization of lipase production

The optimization procedure consisted of two primary stages. Initially, the selection of key PBD components was carried out to determine their optimal levels through CCD and associated contour maps. Finally, the model’s adequacy was confirmed by employing computational statistical analysis (ANOVA) to assess the determination coefficient (*R*^2^) [18, 20–23].

#### Optimizing lipase production using response surface methodology central composite design

Response Surface Methodology (RSM) is a widely recognized statistical model that can be utilized to optimize experimental procedures by assessing the impacts of various parameters and their interactions. In this study, the Design Expert software (Version 7, Stat-Ease Inc., USA) was employed to generate and visualize response surface graphs for lipase production. The focus was on establishing RSM using Central Composite Design (CCD) and optimizing four variables; tween 80, peptone, olive oil concentration, and to enhance lipase production using *P. aeruginosa* while keeping other variables constant. The experimental design matrix consisted of five levels (0, +1, −1, +2, −2) representing the central level, first high level, first low level, second high level, and second low level, respectively. A total of 86 trials were conducted to improve the process parameters. The results were evaluated using the coefficient of determination (*R*^2^), analysis of variance test, and contour response plots. To match the experimental results and identify the relevant model terms, a second-order polynomial equation was developed as the most preferred choice.

$${\text{Y}} =\upbeta 0\,\Sigma\upbeta {\text{iXi + }}\Sigma\upbeta {\text{iXi}}\upbeta {\text{ij + }}\Sigma {\text{XiXj}}$$

Given that Y is the anticipated outcome.

β0, bi, and bij represent the fixed regression coefficients of the mode.

The independent variables are indicated by Xi and Xj.

The experimental design enables the examination of linear, quadratic, and cross-product effects of these parameters, along with the inclusion of replication center points. The experiments were carried out by considering the variables within the expected conditions of the model to ensure the validity of both the model and its results [18, 20–23].

### Purification of lipase enzyme

#### Ammonium sulfate precipitation, dialysis and purification with Sephadex G-100

The culture broth was subjected to centrifugation at 10,000 rpm for 15 min at 4 °C, resulting in the collection of a clear supernatant. To concentrate the enzyme protein, the clear supernatant was then condensed under a vacuum in a rotary evaporator. Undesirable proteins were subsequently precipitated from the condensed broth using a water bath at 45 °C for 30 min. The resulting precipitate was removed, and the enzyme-containing supernatant was cooled. Following Dixon and Web’s approach in 1964, the crude enzyme (supernatant) underwent ammonium sulfate precipitation. Different saturation levels of (NH_4_)_2_SO_4_ (20, 40, 60, and 80%) in 0.01 M sodium phosphate buffer (pH 6.5) were used for lipase precipitation. The crude enzyme solution was gradually supplemented with the appropriate quantities of (NH_4_)_2_SO_4_, and it was then immersed in an ice salt bath for 10 min while being constantly stirred.

These fractions were then centrifuged at 10,000 rpm for 15 min at 4 °C to recover the precipitated protein. Following centrifugation, the sediment fraction containing the protein was dissolved in 10 ml of 0.01 M Na_3_PO_4_ buffer (pH 6.5), and the supernatant was disposed.

The best fraction solution underwent membrane filtration using Millipore dialysis bags (Amicon company) with 10,000 or 12,000 MW cut-off membranes. Dialyzing the sealed dialysis bags against phosphate buffer required constant stirring and intermittent buffer changes during the course of the night, until the salt ions were eliminated.

The dialyzed samples were then filtered using a Millipore filter, and the obtained fractions were redissolved in the same buffer. Enzyme activity was tested using the usual DNS method, and the protein content was measured using the Lowry method with BSA as a reference. The subsequent purification stages utilized the best fraction, where Ammonium sulfate (Ammonium sulfate, A2939-500G, SIGMA-ALDRICH, SLS) was used to load the fraction onto a Sephadex G-100 column [24].

#### Purification with Sephadex G-100

The first step is Sephadex Preparation Hydrate and swell Sephadex G-100 in distilled water or buffer for 24 h then Degas the slurry under vacuum to remove trapped air bubbles, the second is Packing the Column Pack the Sephadex G-100 into the chromatography column without introducing air bubbles then Equilibrate the column by washing with several column volumes of buffer, the third is Sample Loading and Elution Load the dialyzed lipase solution (after ammonium sulfate precipitation) onto the column. Use a small sample volume to maximize resolution then elute the column with the buffer at a constant flow rate (1.0 ml/min) then Collect fractions in tubes (e.g., 2 ml per fraction), last step is, Monitoring by Measuring the protein concentration of fractions using absorbance at 280 nm. Perform a lipase activity assay as mentioned before (e.g., using olive oil as a substrate) to identify active fractions [25].

### Molecular weight determination of the purified lipase enzyme

#### SDS polyacrylamide gel electrophoresis (SDS–PAGE)

SDS–PAGE (Sodium Dodecyl Sulfate Polyacrylamide Gel Electrophoresis) with a 10% T gel (total acrylamide content in the polymerized gel) was used to assess the purity of the lipase enzyme from *P. aeruginosa* isolate following Lammli’s (1970) method. The stock solutions were prepared as follows: acrylamide-bisacrylamide solution, undiluted TEMED (Tetramethylethylenediamine), ammonium persulfate (1.5%), undiluted 2-mercaptoethanol, resolving gel buffer (Tris–HCl [Tris(hydroxymethyl)aminomethane hydrochloride], pH 8.8), stacking gel buffer (Tris–HCl [Tris(hydroxymethyl)aminomethane hydrochloride], pH 6.8), and resolving buffer (10 × , pH 8.6). All solutions were stored at 4 °C until use [26].

### Lipase enzyme stability testing

#### Heat stability

The lipase fraction’s heat stability was assessed by exposing the purified enzyme to different temperatures (ranging from 25 to 60 °C) for 1 h. Subsequently, the remaining lipolytic activities were measured by utilizing olive oil as the substrate [24].

#### pH stability

The purified enzyme was subjected to incubation with various pH buffers for 1 h. The reaction mixtures were incubated following the standard assay protocol, and subsequently, the remaining lipolytic activities were assessed by utilizing olive oil as the substrate [24].

### Evaluation of lipase for anticancer activity

#### Safe concentration range of lipase on Vero cell line isolated from green monkey kidney

The MTT test [27, 28] was used to determine the cytotoxicity of the medications against Vero cells. In summary, a semi-confluent layer was formed by incubating cells in a 96-well microplate at a density of 5 × 10^3^ cells/well in 100 µl complete growth media for an entire night at 37 °C and 5% CO_2_. Drugs were diluted serially and applied to the cells for 48 h at final concentrations of 156, 100, 70, 50, 30, 10, 0.1 U/ml. Instead of adding the tested medications, Dulbecco’s Modified Eagle Medium (DMEM) supplemented with 10% heat-inactivated fetal bovine serum, 100 mg/ml streptomycin, and 100 U/ml penicillin in a humidified 5% (v/v) CO_2_ atmosphere at 37 °C was supplied to the untreated cells (negative control). Complete growth media was substituted for the medications that were evaluated. Following the incubation period, the medium was discarded, and cells were cleaned with PBS. Next, 100 µl of MTT (0.5 mg/ml) was added to each well and incubated for 4 h after being dissolved in media containing serum (DMEM). After mixing and adding 100 µl of DMSO to each well, the formazan crystals were dissolved. Using a microplate reader (Model 4300; Chromate Instrument, Awareness technologies, Inc., Palm City, USA), the optical density (OD) of each well was measured at 492 nm and a reference wavelength of 630 nm. The formula for calculating the percentage of cell viability was (OD test/OD control) × 100. The IC50 value of tested samples (concentration of sample causing a 50% loss of cell proliferation of the vehicle control) was calculated from a four-parameter equation logistic curve (log concentration vs. %cell growth as compared to control cells) using Sigma Plot software version 11.

$${\text{Viability}} = {\text{absorbance}}\,{\text{of}}\,{\text{drug}}/{\text{absorbance}}\,{\text{of}}\,{\text{control}} \times 100$$

$${\text{Cytotoxicity}} = 100 - {\text{viability}}$$

#### In vitro cytotoxic effect of lipase enzyme on HepG-2 cells

The cytotoxicity of lipase against HepG2 cells was determined using the MTT assay [27, 28]. For the purpose of creating a semi-confluent layer, cells were seeded at a density of 5 × 10^3^ cells/well in 100 µl of complete growth media in a 96-well microplate. The cells were then incubated at 37 °C and 5% CO_2_ for the entire night (156, 100, 70, 50, 30, 10, 0.1 U/ml) were the final concentrations of the medicines used to treat the cells over the course of 48 h. Instead of the agent under test, full growth media was supplied to the untreated cells (negative control). Complete growth media was substituted for lipase that were evaluated. Following the incubation period, the medium was disposed of and the cells were cleaned with PBS. Next, 100 µl of MTT (0.5 mg/ml) was added to each well and incubated for 4 h after being dissolved in media containing serum. After mixing and adding 100 µl of DMSO to each well, the formazan crystals were dissolved. Using a microplate reader (Model 4300; Chromate Instrument, Awareness technologies, Inc., Palm City, USA), the optical density (OD) of each well was measured at 492 nm and a reference wavelength of 630 nm. The percentage of viable cell was calculated as (OD test/OD control) × 100. The IC_50_ value of tested samples (concentration of sample causing a 50% loss of cell proliferation of the vehicle control) was calculated from a four-parameter equation logistic curve (log concentration vs. %cell growth as compared to control cells) using Sigma Plot software version 11.

#### Apoptosis and necrosis analysis using flow cytometry

Control and treated HepG-2 cells (as previously described) were subjected to flow cytometry for analysis of apoptosis and necrosis of cell populations using Annexin V- FITC apoptosis discovery tackle (Abcam Inc., Cambridge Science Park, Cambridge, UK) coupled with 2 fluorescent channels flowcytometry. After treatment with test composites for the specified duration, cells (10^5^ cells) are collected by trypsinization and washed doubly with ice-cold PBS (pH 7.4). Also, cells are incubated in dark with 0.5 ml of Annexin V- FITC/PI result for 30 min in dark at room temperature according to manufacturer protocol. After staining, cells are fitted via ACEA Novocyte™ inflow cytometer (ACEA Biosciences Inc., San Diego, CA, USA) and anatomized for FITC and PI fluorescent signals using FL1 and FL2 signal sensor, independently (λex/em 488/530 nm for FITC and λex em 535/617 nm for PI). For each sample, 12,000 events are acquired and positive FITC and/or PI cells are quantified by quadrant analysis and calculated using ACEA NovoExpress™ software (ACEA Biosciences Inc., San Diego, CA, USA) [29–32].

#### Real time PCR for analysis of *BCL-2* gene

Total RNA was extracted from both control and treated HepG-2 cells (as previously described) using a RNeasy mini kit (Cat. No. 74104, Qiagen, Hilden, Germany) according to the manufacturer’s instructions. The concentration and quality of the RNA was determined using a Plate reader FLUOstar Omega (BMG LABTECH) Spectrophotometer (A260/280 ratio). The isolated RNA was reverse transcribed into cDNA using GScript First-Strand Synthesis Kit (Cat. No.: MB305-0050, Gene DireX, Taiwan) according to the manufacturer’s guidelines. Gene expression levels were determined using SYBR Green qPCR master mix (Xpert Fast SYBR (uni), Cat. No. # GE20.100, Porto, Portugal) and the Bio-Rad platform (Bio-Rad CFX OPUS 96) in a total volume of 20 µl consisting of 2 µl cDNA, 2 µl (0.3–0.5 µM) forward and reverse primers, 10 µl master mix, and 6 µl nuclease free water [33].

## Results and discussion

### Isolation and identification of lipase-producing bacteria

**Fig. 1:**
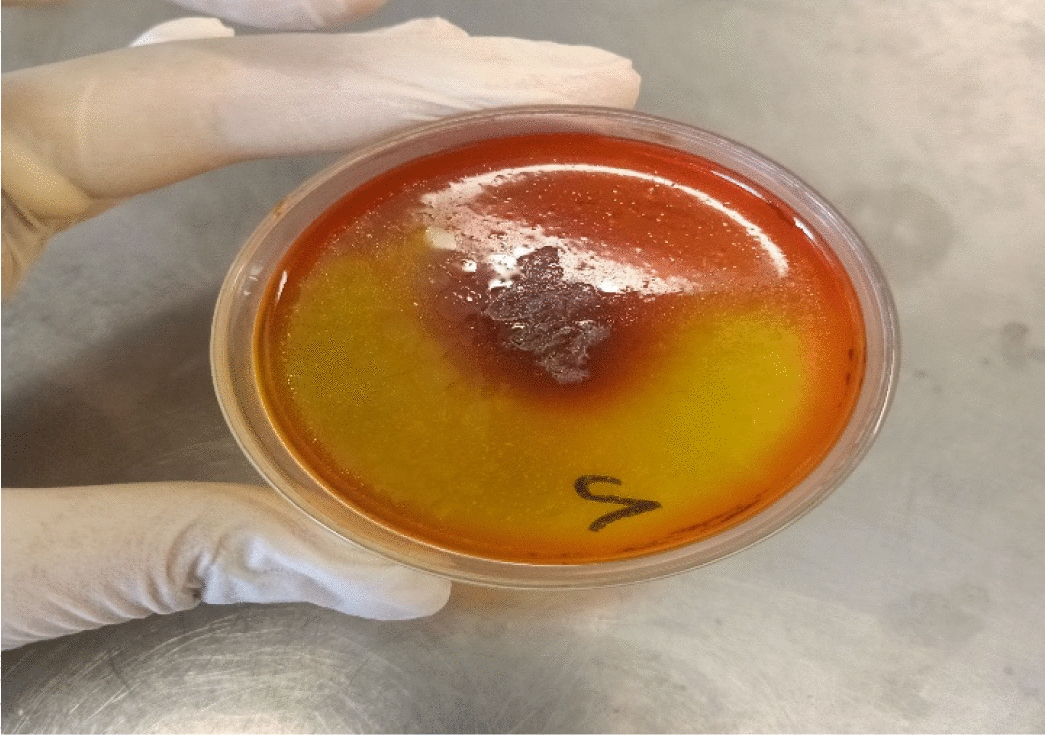
Phenol red agar test of isolated *P. aeruginosa* showing yellow zone around colonies

Fifty-one pure bacterial isolates were recovered from the biological specimens from different human sources (blood, urine, sputum, wound pus) being investigated, as previously described in the “Materials and methods” section. These isolates were tested for lipase activity using phenol red agar media. Out of the 51 isolates, only 7 were found to exhibit lipase production capabilities. Among these seven isolates, the one displaying the largest hydrolysis zone was selected for further analysis. During the initial screening of the bacterial isolates, it was observed that isolate number 2 obtained from wound pus, belonging to the *Pseudomonas* genus, demonstrated the ability to produce lipase enzyme using the phenol red agar plate method (Fig. 1). The isolate with the largest yellow zone (24 mm) was chosen for further investigation to quantify the lipase activity (Table 1). In comparison, Elazzazy and Fawzy (2021) used phenol red agar plates to screen for lipase-producing bacteria, the strain *Lysinibacillus* PL33 exhibited the highest lipolytic activity, producing a yellow zone with a diameter of 22 mm [34].

**Table 1:** Zone diameters in mm of lipase-producing *P. aeruginosa* isolates grown on phenol red agar medium

Isolate code	Zone diameters (mm ± SD)*
P1	21 ± 1.74
P2	**24 ± 1.74**
P3	22 ± 1.74
P4	19 ± 1.74
P5	11 ± 1.74
P6	22 ± 1.74
P7	15 ± 1.74

*Bold values refere to the highest lipase-producing bacteria

**Fig. 2:**
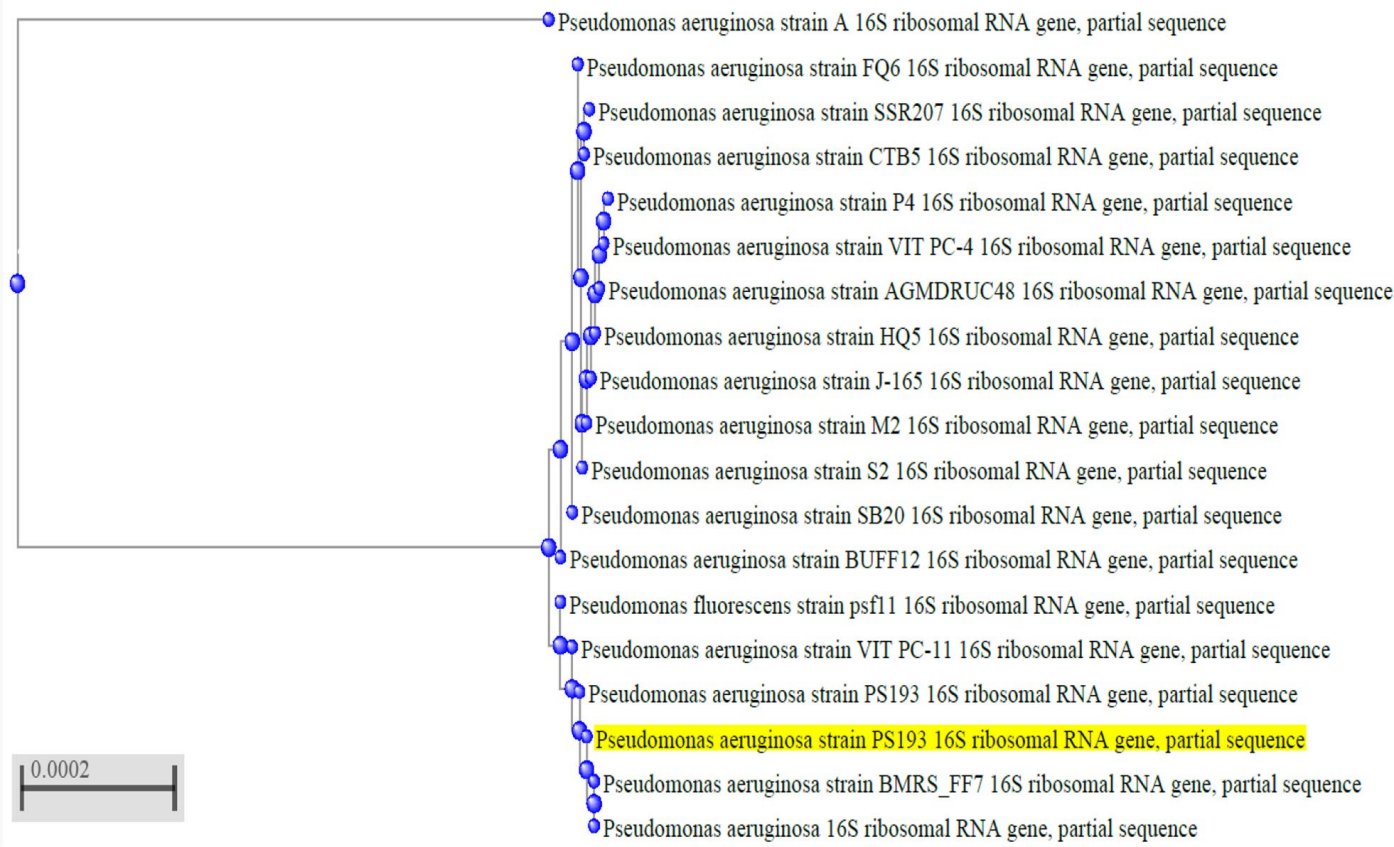
Phylogenetic analysis of *Pseudomonas aeruginosa* isolates depending on 16s RNA sequence homology using BLASTn

Isolate number 2, which exhibited the highest lipase activity, this isolate was identified as a motile, Gram-negative, rod-shaped bacterium. Based on its morphological and biochemical characteristics, as detailed in Table 2, it was further confirmed as *Pseudomonas aeruginosa*. Standard biochemical tests, as outlined in Bergey’s Manual of Determinative Bacteriology, were performed to confirm its identity. A comparison of the 16S ribosomal RNA gene sequence (395 base pairs) with closely related sequences in the NCBI database revealed a 99% sequence similarity with *P. aeruginosa* strain CUAB-ODEDELE02. Phylogenetic analysis using the neighbor-joining method showed that the isolate belongs to the *Pseudomonas* genus and is closely related to *P. aeruginosa* strains (Fig. 2). The nucleotide sequence of the *P. aeruginosa* isolate has been deposited in the GenBank database under the accession number PP436388 (http://ncbi.nlm.nih.gov/PP436388) [24].

**Table 2:** Morphological and biochemical characters of isolated organism

Feature	Results
Characters on nutrient agar	Greenish color
Gram staining/morphology under a microscope	Negative, rods
Catalase	Positive (+ve)
Citrate	Positive (+ve)
Gelatin hydrolysis	Positive (+ve)
Cetrimide	Positive (+ve)
Methyl red	Negative (−ve)
Indole	Negative (−ve)
VP	Negative (−ve)
Oxidase	Positive (+ve)
Carbohydrate fermentation	Negative (−ve)
Nitrate reduction	Positive (+ve)
Motility	Motile (unipolar)
Pigment	Positive (+ve)

### Lipase assay

Lipase-positive strains were determined based on the yellow zone around bacterial colonies grown on phenol-red agar. They were subjected to lipase assay and data presented in Table 3 showed that only 3 strains exhibited the highest productivity for lipase enzyme. The three strains recorded a lipase activity ranging between 89.84 and 167.96 U/ml. In the study of Oyeleke and Adedeji (2015), *P. aeruginosa* demonstrated a maximum lipase activity of 42 U/ml at pH 7.0. Interestingly, our study observed significantly higher lipase activity (approximately 4 times), with the strain exhibiting a value of 167.96 U/ml. This notable difference in lipase production may be attributed to variations in the experimental conditions, such as the strain used, medium composition, or environmental factors [35].

**Table 3:** Lipase activity in U/ml for phenol red positive *P. aeruginosa* strains

Strain code	Lipase activity (U/ml)
P1	140.62
P2	167.96
P3	89.84
P4	122.21
P5	143.52
P6	125.75
P7	96.54

### Mutagenesis

**Fig. 3:**
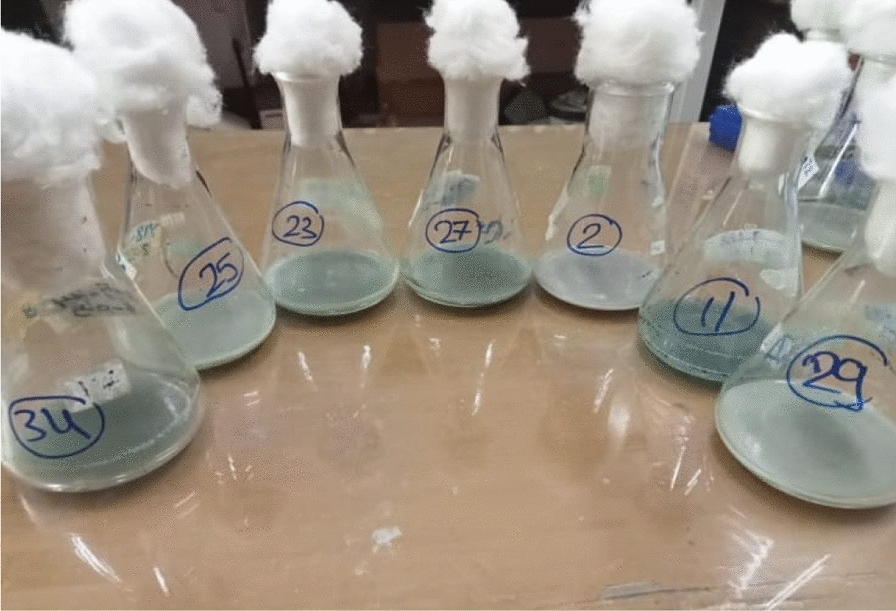
Mutant strains of *P. aeruginosa* and the wild type with the highest lipase activity that grown in production media

There are 30 surviving colonies resulting from mutation study and only six mutants (Fig. 3) displayed high lipase activity (Table 4). Our findings showed that one mutant demonstrated the highest activity, recording a value of 312.09 U/ml. In our study, lipase activity increased by 85.87%, from 167.96 to 312.09 U/ml after mutation, indicating a significant enhancement in enzyme performance. Similar findings were reported by Ouellet et al. (2015) where a 50% increase in enzyme activity was observed after a single round of random mutagenesis [36].

**Table 4:** *P. aeruginosa* mutants that displayed high lipase activity following mutagenesis study

Mutant code*	Lipase enzyme (U/ml)
PM1	307.8
PM2	198.6
PM3	241.7
PM4	302.5
PM5	185.9
PM6	312.09

*PM refere to mutants of *P. aeruginosa*

### Determination of optimal basal conditions for lipase production (un-optimized conditions) by OFAT protocol

#### The influence of various carbon sources on lipase production

**Fig. 4:**
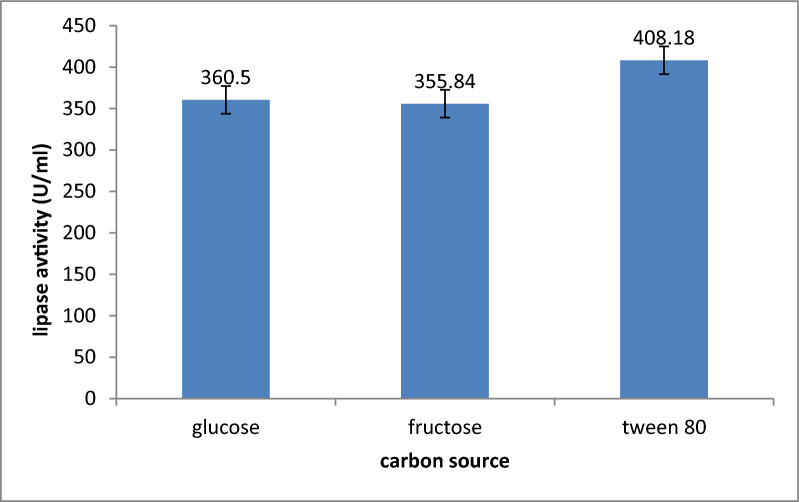
Effect of different carbon sources on lipase activity

Tween 80 emerged as the most effective carbon source because it stimulated lipase biosynthesis and secretion as it increases cell permeability and facilitates lipase excretion across the cell membrane [34], with a mean yield after triplicate experiment of 408.18 U/l, glucose (360.5 U/ml) and fructose (355.84 U/ml) as illustrated in Fig. 4. Furthermore, subsequent research findings indicated that distinct organisms exhibit varying abilities in utilizing carbon sources for the biosynthesis of bacterial enzymes.

#### The impact of various nitrogen sources on the production of lipase

**Fig. 5:**
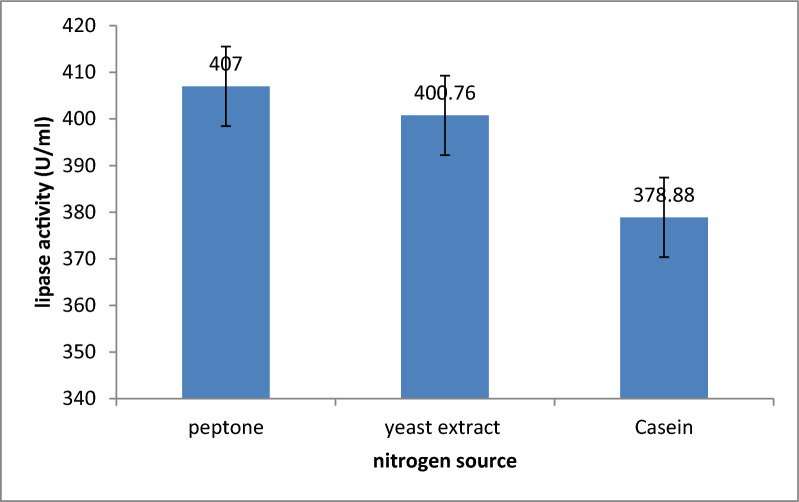
Effect of different nitrogen sources lipase activity

The effect of different nitrogen sources on lipase production was evaluated. Organic sources play a vital role in lipase production, among the different nitrogen sources tested, lipase showed the best activity with the utilization of peptone as a nitrogen source with three replicates as follows: by average activity of 407 U/ml; SD = 2.52 followed by yeast extract with average activity of 400.76 U/ml; SD = 0.18 and Casein with average activity of 378.88 U/ml; SD = 0.21 as shown in Fig. 5. The capability of utilizing the fermentation media components with different nitrogen sources affects enzymatic production which differs from organism to organism. Other research findings showed that each microorganism has its specific culture conditions for maximum lipase production [37]. Kanimozhi and Perinbam (2010) found that peptone was the most effective nitrogen source, with lipase activity of 41.5 U/ml, followed by yeast extract and casein. In our study, peptone showed significantly higher lipase activity (407 U/ml), followed by yeast extract (400.76 U/ml) and casein (378.88 U/ml). This difference suggests better optimization of experimental conditions in our work. Peptone’s role in providing essential amino acids and peptides is critical for enhancing lipase production [38].

#### Optimizing lipase activity through response surface methodology (RSM)

The statistical and mathematical analysis of multivariable data obtained from response surface methodology (RSM) is crucial to improving and optimizing lipase production by *P. aeruginosa* mutant. In this study, a central composite model was utilized for lipase production using an 86-run experimental design on mutant *P. aeruginosa*. The matrix consisted of four factors with three levels (−1, 0, and +1) and three replicates at the central point. Table 5 presents the independent variables with a coded matrix, of responses. The alteration in enzyme activity was observed during the 86 runs of the experiment due to the varying conditions in each run, emphasizing the importance of statistical optimization of fermentation conditions over traditional methodology. The optimized culture condition for maximum lipase activity was achieved at pH 9, 24-h incubation time, 2.5% tween 80, and 2.5% peptone, resulting in an activity of 499.33 U/mg.

**Table 5:** Design of different trials of the RSM for independent variables and responses by mutant *P. aeruginosa*

Run	pH	Temp	Tween 80	Peptone	Olive oil conc	Incub. time	Response (U/ml)
1	9	55	0.5	0.5	3	72	496.25
2	7	37.5	1.5	1.5	3	48	411.26
3	7	37.5	1.5	1.5	2	48	410.08
4	9	55	0.5	2.5	3	72	495.37
5	9	55	2.5	2.5	3	24	496.41
6	9	20	0.5	0.5	3	24	497.43
7	5	20	2.5	2.5	3	72	363.22
8	5	55	0.5	0.5	1	24	361.92
9	5	55	2.5	0.5	3	72	366.45
10	5	55	2.5	2.5	1	24	364.56
11	7	37.5	1.5	1.5	2	48	413.49
12	5	20	2.5	2.5	1	24	366.46
13	9	55	0.5	2.5	3	24	491.23
14	9	20	2.5	2.5	1	72	497.44
15	7	37.5	1.5	1.5	2	48	412.39
16	5	20	2.5	0.5	1	24	361.21
17	9	55	0.5	0.5	1	72	493.39
18	9	55	2.5	0.5	1	24	490.21
19	5	55	2.5	0.5	1	72	365.38
20	7	37.5	1.5	1.5	2	48	414.47
21	9	37.5	1.5	1.5	2	48	494.01
22	9	55	2.5	0.5	3	24	496.49
23	5	20	0.5	0.5	3	24	367.48
24	9	20	0.5	0.5	3	72	496.84
25	7	37.5	1.5	1.5	2	48	416.95
26	5	55	2.5	2.5	3	72	363.49
27	9	20	2.5	0.5	3	24	492.56
28	5	20	2.5	2.5	3	24	368.49
29	9	55	0.5	0.5	3	24	496.21
30	5	20	0.5	2.5	1	24	363.33
31	7	37.5	1.5	1.5	2	48	414.46
32	9	20	2.5	2.5	3	72	491.26
33	5	37.5	1.5	1.5	2	48	365.53
34	9	55	2.5	0.5	3	72	490.47
35	7	37.5	1.5	1.5	2	48	413.22
36	9	55	0.5	2.5	1	24	493.56
37	9	20	2.5	0.5	1	24	495.31
38	5	20	2.5	2.5	1	72	366.49
39	5	55	2.5	2.5	1	72	363.26
40	7	37.5	0.5	1.5	2	48	415.53
41	7	37.5	1.5	1.5	2	48	410.31
42	5	20	0.5	0.5	1	72	367.74
43	9	20	2.5	2.5	3	24	493.29
44	9	55	2.5	0.5	1	72	495.42
45	7	20	1.5	1.5	2	48	409.37
46	5	55	2.5	0.5	1	24	368.46
47	9	55	2.5	2.5	1	24	490.77
48	5	55	2.5	2.5	3	24	362.26
49	9	55	2.5	2.5	3	72	491.32
50	7	37.5	2.5	1.5	2	48	414.54
51	5	20	0.5	2.5	1	72	365.88
52	7	55	1.5	1.5	2	48	412.11
53	9	20	0.5	2.5	3	72	494.45
54	9	20	0.5	2.5	1	24	491.59
55	9	55	0.5	2.5	1	72	490.29
56	7	37.5	1.5	1.5	2	72	415.83
57	9	55	2.5	2.5	1	72	498.25
58	7	37.5	1.5	0.5	2	48	414.01
59	9	20	0.5	2.5	3	24	492.36
60	5	55	2.5	0.5	3	24	366.11
61	7	37.5	1.5	1.5	2	48	413.08
62	5	20	2.5	0.5	1	72	362.33
63	5	20	0.5	2.5	3	24	364.39
64	7	37.5	1.5	1.5	2	24	419.41
65	9	20	0.5	0.5	1	24	495.39
66	5	55	0.5	0.5	3	72	369.87
67	5	20	2.5	0.5	3	72	360.55
68	5	20	0.5	0.5	1	24	361.22
69	5	20	0.5	0.5	3	72	365.45
70	5	55	0.5	2.5	1	72	366.93
71	9	20	0.5	2.5	1	72	493.89
72	5	20	0.5	2.5	3	72	367.71
73	5	55	0.5	0.5	1	72	365.34
74	9	20	2.5	2.5	1	24	499.33
75	7	37.5	1.5	1.5	1	48	414.39
76	7	37.5	1.5	1.5	2	48	410.99
77	9	20	2.5	0.5	1	72	497.72
78	5	55	0.5	2.5	3	72	362.74
79	7	37.5	1.5	2.5	2	48	411.13
80	5	55	0.5	2.5	3	24	364.44
81	9	20	0.5	0.5	1	72	496.37
82	9	55	0.5	0.5	1	24	495.52
83	5	55	0.5	0.5	3	24	367.72
84	9	20	2.5	0.5	3	72	493.24
85	5	20	2.5	0.5	3	24	360.84
86	5	55	0.5	2.5	1	24	365.98

(Run 74). The determination coefficient (*R*^2^) showed a high accuracy of the model, with a predicted value *R*^2^ of 0.9976 is in reasonable agreement with the Adjusted *R*^2^ of 0.9984

##### The model validation

The proposed model’s validity was assessed by predicting the lipase production of bacteria mutants for each trial in the matrix. The experimental results in Table 5 showed that the maximum observed lipase production (499.33) in run 74. The analysis of variance results for lipase production by bacteria mutants is presented in Tables 5 and 6. The model was highly significant.

**Table 6:** Regression values by CCD

Std. dev	2.29	*R*^2^	0.9989
Mean	425.86	Adjusted *R*^2^	0.9984
C.V. %	0.5386	Predicted *R*^2^	0.9976
		Adeq precision	103.6701

##### ANOVA for quadratic model

Factor coding is Coded (Table 7).

**Table 7:** Response 1: Enzyme activity

Source of variation	Sum of squares	Degrees of freedom	Mean squares	*F*-value	*P* value	Significance
Model	2.806E+05	27	10,392.92	1975.41	<0.0001	Significant
A-pH	2.763E+05	1	2.763E+05	52,518.83	<0.0001	
B-Temp	0.0909	1	0.0909	0.0173	0.8959	
C–C. Source	6.19	1	6.19	1.18	0.2824	
D-N. Source	5.66	1	5.66	1.08	0.3039	
E-olive oil conc	0.8937	1	0.8937	0.1699	0.6818	
F-Inc. time	1.14	1	1.14	0.2175	0.6427	
AB	13.53	1	13.53	2.57	0.1142	
AC	5.02	1	5.02	0.9548	0.3326	
AD	5.98	1	5.98	1.14	0.2906	
AE	3.06	1	3.06	0.5813	0.4489	
AF	0.2082	1	0.2082	0.0396	0.8430	
BC	0.2930	1	0.2930	0.0557	0.8143	
BD	16.25	1	16.25	3.09	0.0841	
BE	6.08	1	6.08	1.16	0.2867	
BF	0.8860	1	0.8860	0.1684	0.6831	
CD	29.63	1	29.63	5.63	0.0210	
CE	35.63	1	35.63	6.77	0.0117	
CF	9.93	1	9.93	1.89	0.1748	
DE	11.06	1	11.06	2.10	0.1524	
DF	0.4209	1	0.4209	0.0800	0.7783	
EF	14.37	1	14.37	2.73	0.1038	
A^2^	616.44	1	616.44	117.17	<0.0001	
B^2^	20.99	1	20.99	3.99	0.0505	
C^2^	4.23	1	4.23	0.8037	0.3737	
D^2^	3.07	1	3.07	0.5842	0.4478	
E^2^	1.85	1	1.85	0.3511	0.5558	
F^2^	36.62	1	36.62	6.96	0.0107	
Residual	305.15	58	5.26			
Lack of fit	264.81	49	5.40	1.21	0.4074	Not significant
Pure error	40.33	9	4.48			
Cor total	2.809E+05	85				

Sum of squares is Type III—Partial.

The Model *F*-value of 1975.41 implies the model is significant. There is only a 0.01% chance that an *F*-value this large could occur due to noise.

*P* values less than 0.05 indicate model terms are significant. In this case, A, CD, CE, A^2^, and F^2^ are significant model terms. Values greater than 0.1000 indicate the model terms are not significant. If there are many insignificant model terms (not counting those required to support hierarchy), model reduction may improve your model.

**Fig. 6:**
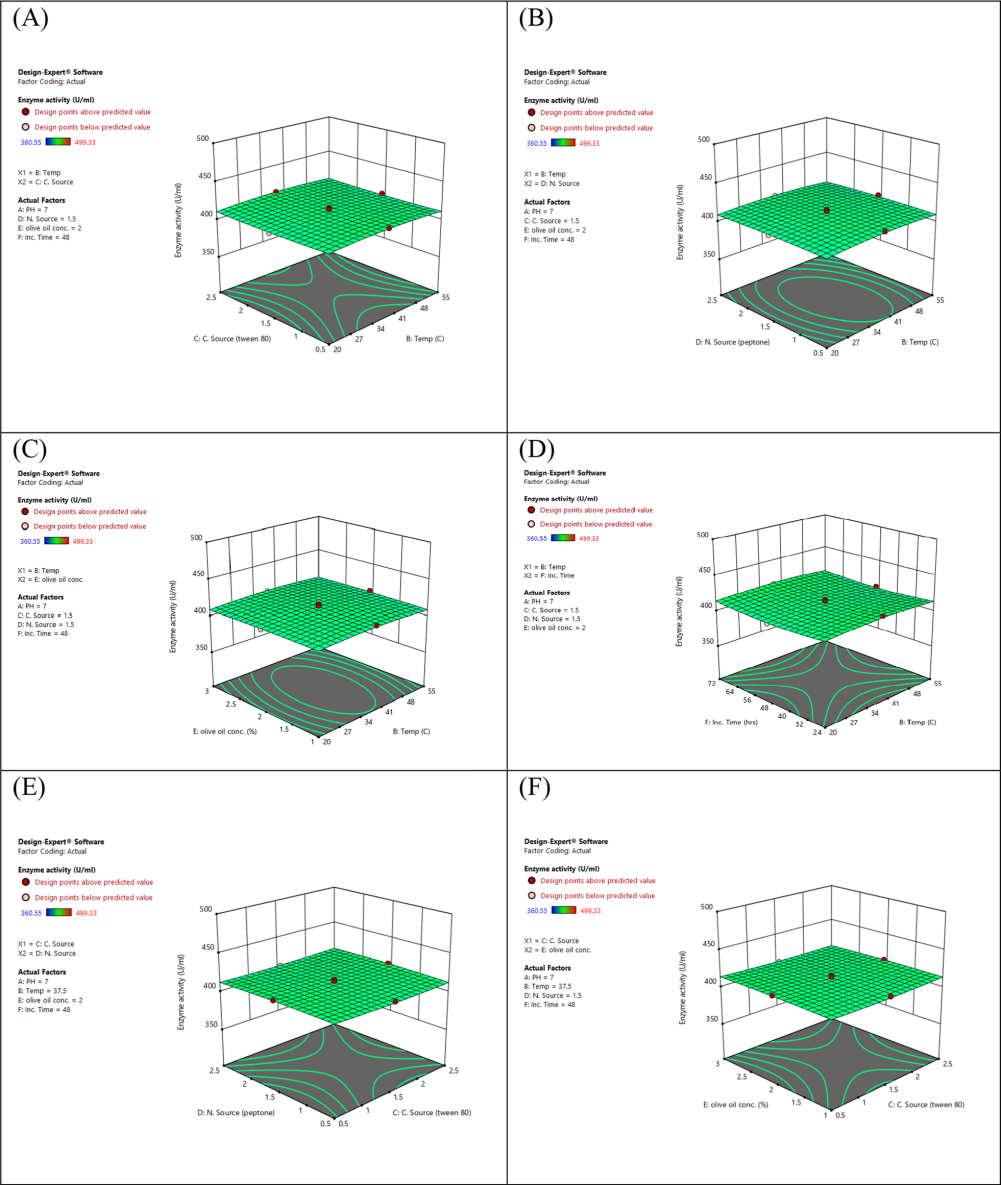

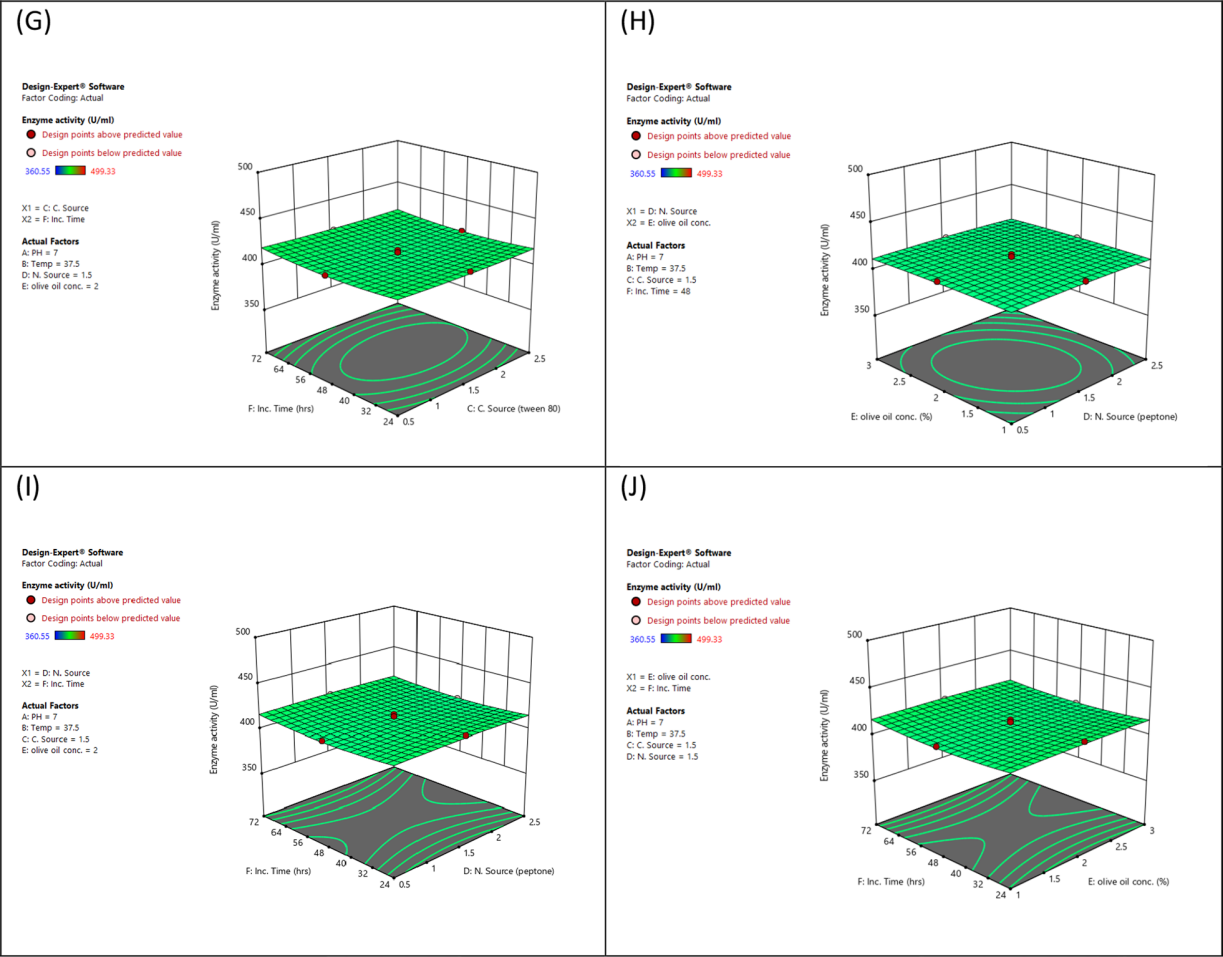
Response surface plot of the interaction effect of **A** temperature, carbon source **B** temperature, nitrogen source **C** temperature, olive oil concentration **D** temperature, incubation time **E** carbon source, nitrogen source **F** carbon source, olive oil concentration **G** carbon source, incubation time **H** nitrogen source, olive oil concentration **I** nitrogen source, incubation time **J** olive oil concentration, time

The Lack of Fit *F*-value of 1.21 implies the Lack of Fit is not significant relative to the pure error. There is a 40.74% chance that a Lack of Fit *F*-value this large could occur due to noise (Fig. 6).

### Purification of lipase enzyme

#### Ammonium sulfate precipitation

Lipase was separated from culture filtrate by centrifugation at 10,000 rpm for fifteen min, and then the collected supernatant was utilized as crude enzyme before selective precipitation with salts such as ammonium sulfate. The highest lipase activity was shown in fractions precipitated at 60% and 40% saturation level of ammonium sulfate, with 337 U/ml and 327.8 U/ml specific activity of lipase, respectively (Table 8). The equilibrium of charges on the protein’s surface and the disruption of the surrounding aqueous layer, which causes the protein to settle, determine the concentration of ammonium sulfate (Table 8). In this study, the highest lipase activity was observed in fractions precipitated at 60% and 40% saturation level of ammonium sulfate, yielding specific activities of 337 U/ml and 327.8 U/ml, respectively. These results are in agreement with that of Oyeleke et al. (2015), who reported 300 U/ml of lipase activity after ammonium sulfate precipitation at a similar saturation level. This confirms ammonium sulfate’s effectiveness in enzyme purification, supporting its use due to its solubility, cost-effectiveness, and ability to stabilize the enzyme [39].

**Table 8:** Purification steps of lipase and its final yield

Purification step	Total protein (mg/ml)	Total activity (U/ml)	Specific activity (U/mg)	Purification fold	Yield (%)
Crude enzyme	15.03	3119	260.21	1	100.0
20–40% Ammonium sulfate	4.82	1720	327.8	1.26	55.15
40–60% Ammonium sulfate	4.60	1580	337	1.29	50.65
Sephadex G-100 chromatography	4.26	735	1123	4.32	23.57

#### Dialysis against phosphate buffer

The obtained ammonium sulfate precipitate was centrifuged and introduced into membrane filtration using a 10,000 MW cut-off dialysis bag overnight against 0.01 M phosphate buffer at (pH = 6.5) with three phosphate buffer adjustments were made to produce a clear dialysate, which was subsequently dissolved in the least quantity of phosphate buffer to measure the activity of the enzyme. The enzyme assay was carried out using the standard parameters previously mentioned, and the specific activity was achieved using a purification fold as indicated in Table 8.

#### Loading on Sephadex G-100

Ammonium sulfate (Ammonium sulfate, A2939-500G, SIGMA-ALDRICH, SLS) was used to load fraction onto Sephadex G-100 column (Sephadex G-100, 100 g, 17006001, cytiva). The lipase was eluted from the column at a flow rate of (1.0 ml/min) (Sharma et al., 2017).

#### Determination of molecular weight of lipase by SDS–PAGE gel electrophoresis

**Fig. 7:**
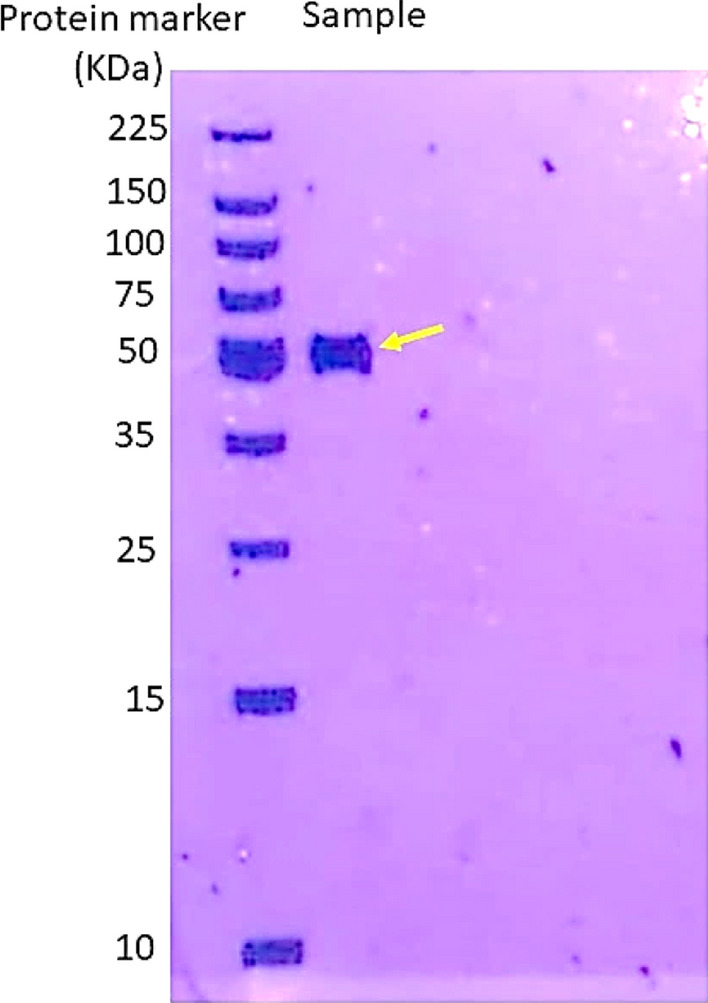
Molecular weight of lipase enzyme from *P. aeruginosa* using SDS–PAGE gel electrophoresis stained with Coomassie Brilliant Blue R-250 showing a band of 53 kDa as pointed by the yellow arrow

Using 7.5% stacking gel and 10% resolving gel, SDS–PAGE was carried out under non-reducing conditions. To make the protein bands visible, they were stained with Coomassie brilliant blue R-250. Results revealed that the molecular weight of the enzyme was specified at 53 kDa (Fig. 7). Similar findings were reported by Nourbakhsh and co-workers [40].

### Characterization of purified lipase

#### pH stability

**Fig. 8:**
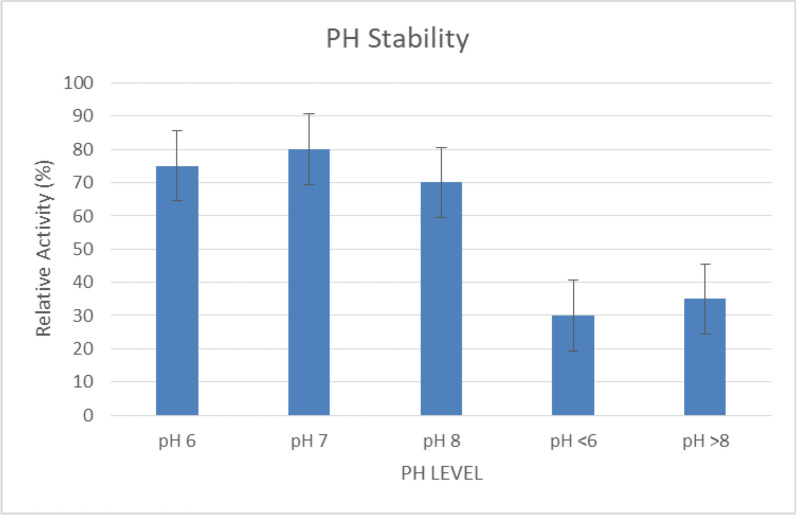
This chart illustrates the stability of lipase at different pH levels, showing maximum activity retention (70–80%) at pH 6–8, with decreased stability outside this range

At pH 6–8, the enzyme retained 70–80% of its activity (Fig. 8). Additionally, below pH 6 or above pH 8, the enzyme showed a decline in activity, with reduced stability at extreme pH values. Therefore, the purified lipase showed optimal stability within a pH range of 6–8, retaining 70–80% of its activity. This effect was in agreement with Abdel-Fattah et al. (2005), who reported stable lipase activity from *P. aeruginosa* at the same pH range.

#### Thermal stability at a temperature range 25–35 °C

**Fig. 9:**
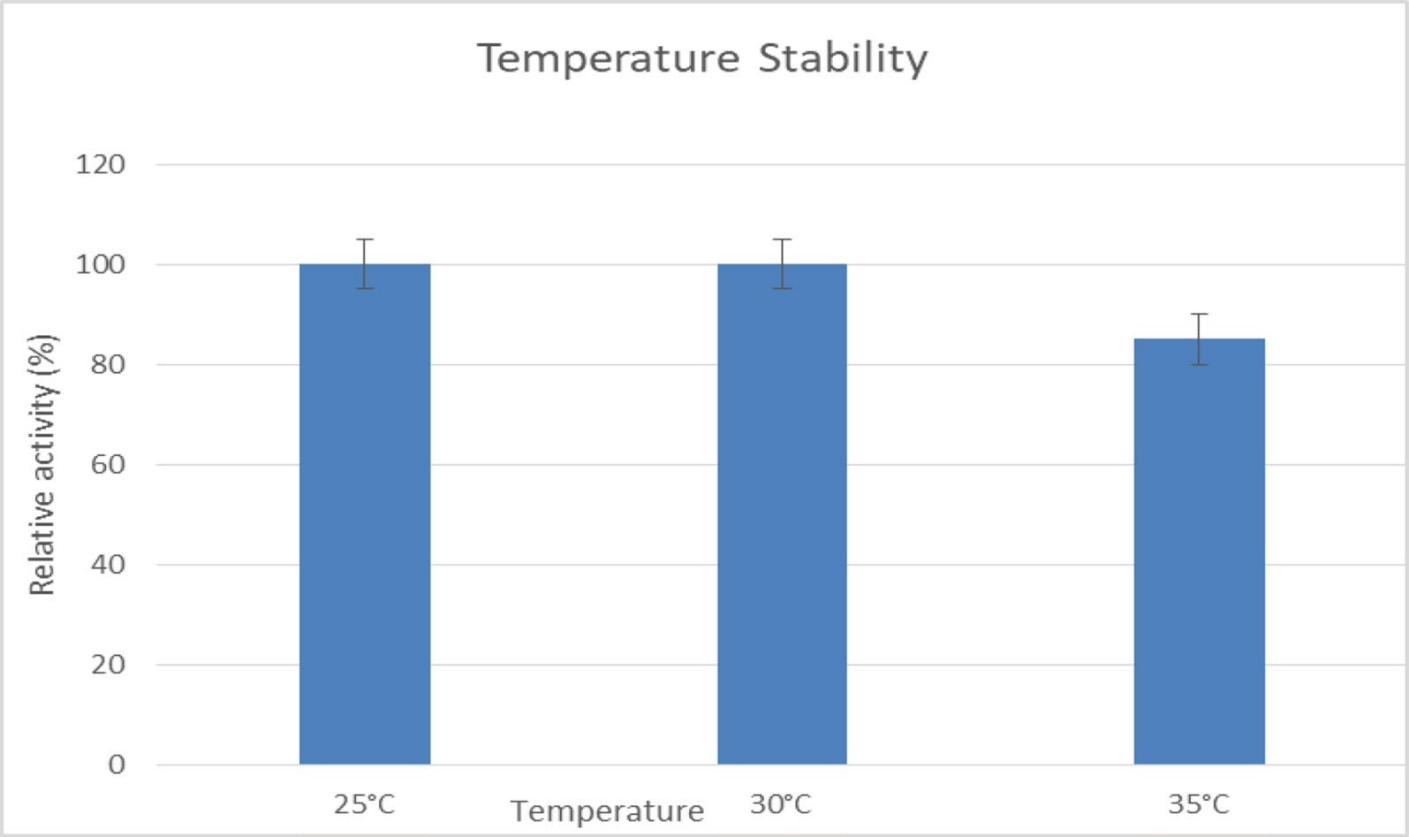
This chart illustrates the stability of lipase at different temperatures, showing maximum activity at (25–30 °C), with decreased stability outside this range

At 30 °C, the enzyme retained 100% of its activity after 1 h, however, at 35 °C, 85% of its initial activity was retained (Fig. 9). Additionally, temperatures above 35 °C likely cause a further decrease in activity. Similarly, Abdel-Fattah et al. (2005) observed optimal activity at 35 °C and stability up to 50 °C. This suggests that lipase remains stable within moderate temperature and pH conditions, supporting its industrial applicability [41].

### Evaluation of lipase as anticancer

#### Evaluation of lipase-safe concentrations using Vero cell line derived from green monkey kidney

**Fig. 10:**
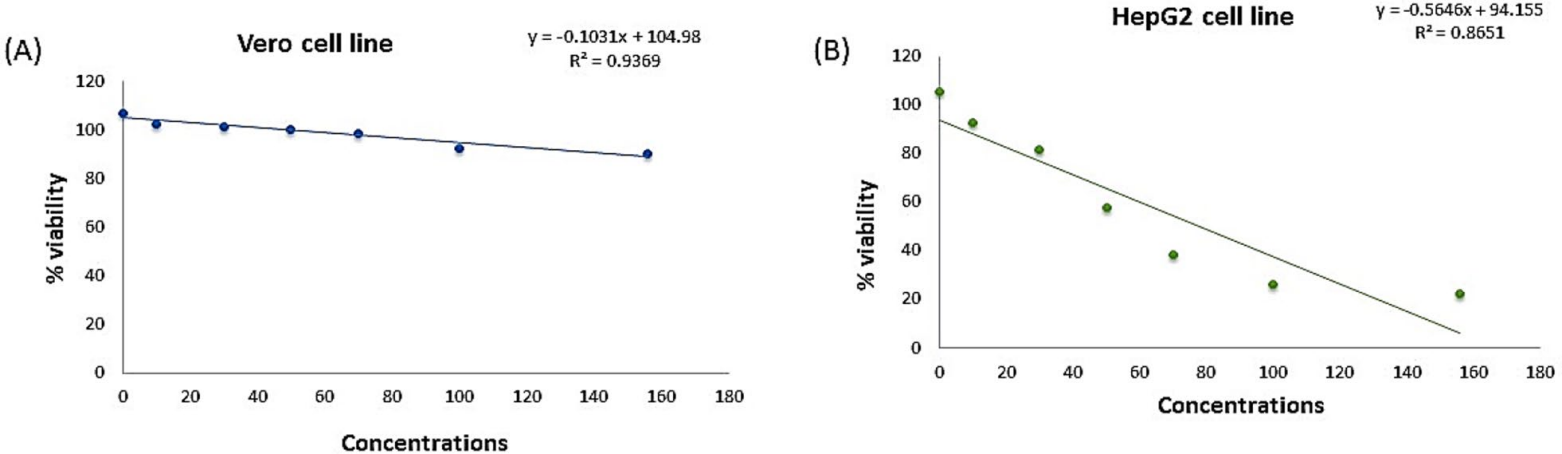
Effect of microbial lipase on the viability of **A** Vero cell line, and **B** HepG2 cell line

For determination of the sub-toxic concentrations of lipase on mammalian cells which will be used later for its useful applications, the Vero cell line was incubated at 37 °C in a humidified incubator with 5% CO_2_ with increasing lipase concentrations, and then the cytotoxic activity was measured by the MTT assay. The viability percentage of Vero cells remained constant (nearly 100%) until reaching a concentration of 533.27 U/ml. These results indicated that lipase was tolerated by the Vero cell line and that lipase can be used as a safe agent in humans at a concentration below 533.27 U/ml (Fig. 10A). Interestingly, our study is the first report that assesses the sub-toxic concentrations of lipase on Vero cells using the MTT assay. Our results show that lipase is well-tolerated up to 533.27 U/ml, with nearly 100% cell viability, suggesting it is safe for human applications at concentrations below this threshold.

#### In vitro cytotoxic effect of lipase

**Fig. 11:**
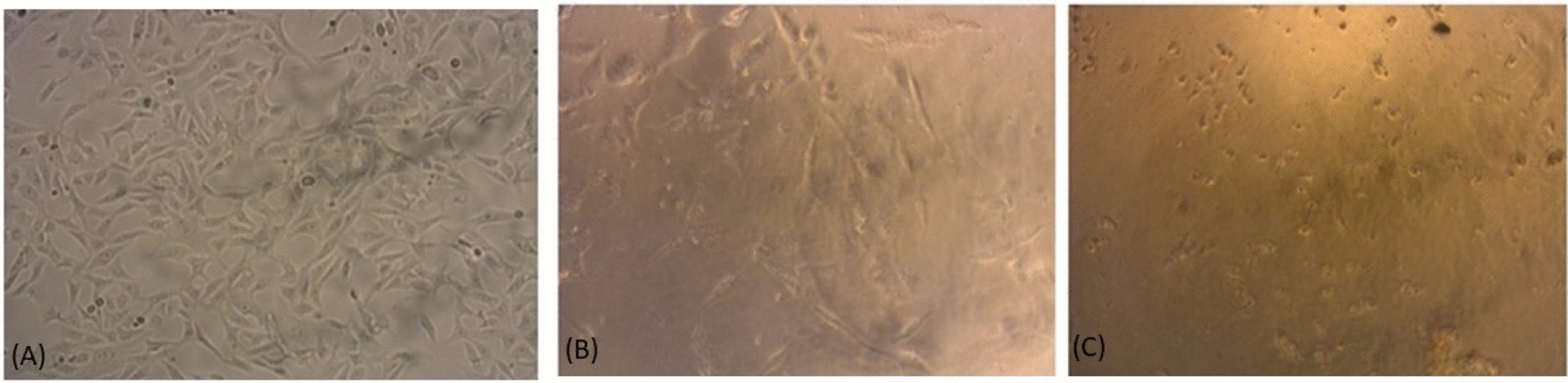
Morphological changes in HepG-2 cell line after 24 h treatment with lipase (IC_50_) for panel (**B**) and (2 IC_50_) for panel (**C**)

The MTT assay was used to estimate the in vitro cytotoxic effect of lipase on the HepG-2 cell line at concentrations below 533.27 µg/ml (Fig. 10B). Data revealed that the concentration of lipase that led to a 50% reduction (IC_50_) in the metabolically active population was 78.21 U/ml as (Fig. 10B). Morphological changes were also noticed on the treated cells that appeared rounded, and floating (Fig. 11B) indicating cell death compared to the untreated control (Fig. 11A) that were seen as fusiform and well connected in addition to their adherence to the container surface (Fig. 11). In this study, lipase showed an IC_50_ of 78.21 U/ml for HepG-2 cells, which is similar to findings by Cheng et al. (2017), who reported an IC_50_ of 70 U/ml in the same cell line. Both studies indicate a dose-dependent reduction in cell viability, supporting the potential anticancer properties of lipase [42].

#### Apoptosis and necrosis analysis using flow cytometry

**Fig. 12:**
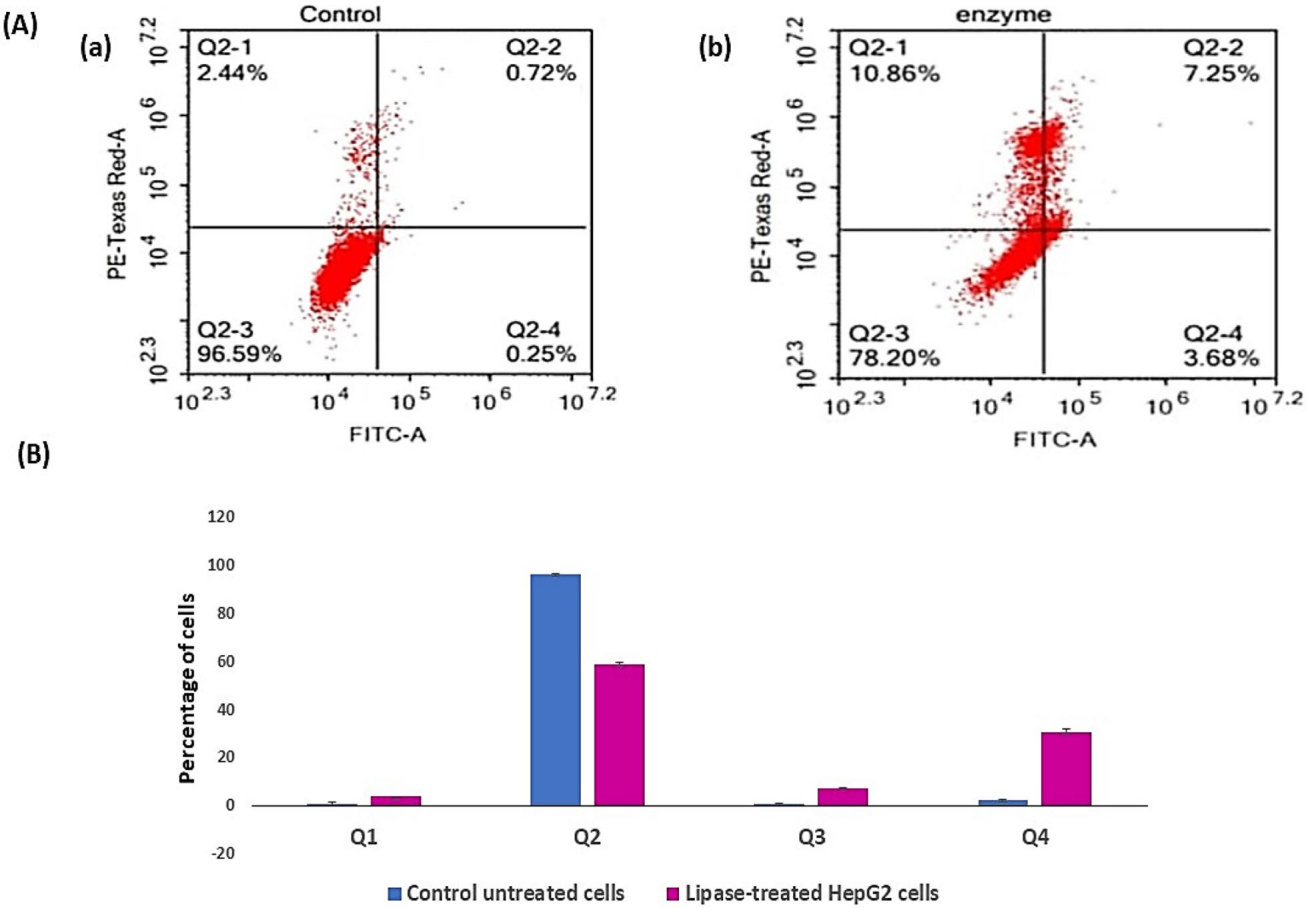
**A** Data of flow cytometry for (a) untreated control Hep-G2 cells and (b) lipase-treated HepG2 showing shift of the cells towards late apoptosis. (Q1) refers to necrosis phase, (Q2) means late apoptosis phase, (Q3) denotes normal intact cells and (Q4) represents early apoptosis phase. **B** Percentages of HepG2 cells distributed in different phases

To further prove whether the inhibition of hepatocellular carcinoma growth after lipase treatment was associated with the induction of apoptosis and/or necrosis. The untreated and treated HepG-2 cells were stained by Annexin V-FITC and PI stains and analyzed by Novocyte Flow Cytometer. As revealed in Fig. 12, the early apoptotic cells increased from 2.27 to 30.37%, more than 13 folds, and the late apoptotic cells increased from 0.69 to 7.24%, more than 10 folds, however, the necrotic cells augmented from 0.71 to 3.68%, more than fivefolds, after treating the cells with lipase at IC_50_. These data confirmed the anticancer activity of microbial lipase against HEpG-2 cells including the stimulation of both apoptosis and necrosis. In the current study, lipase treatment on HepG-2 cells triggered a notable shift in cell fate, with a striking increase in both early and late apoptotic cells, as well as necrotic cells, underscoring its promising anticancer potential. Specifically, early apoptotic cells surged from 2.27 to 30.37%, late apoptotic cells climbed from 0.69 to 7.24%, and necrotic cells rose from 0.71 to 3.68%. These results align with the findings of Chen et al. (2019), who demonstrated similar apoptosis induction in HepG-2 cells using lipase from *Candida antarctica*. In their study, early apoptotic cells increased from 4.5 to 29%, while late apoptotic cells rose from 1.1 to 8% at the IC50 concentration [43].

#### Expression of *BCL-2* gene in presence of lipase and analysis of RT-PCR results

**Fig. 13:**
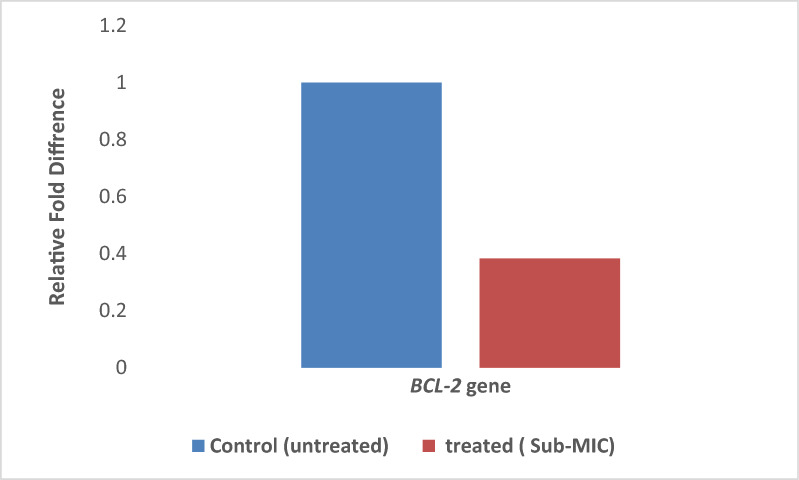
Fold reduction in the expression of *Bcl-2* gene following the treatment with lipase enzyme. GAPDH gene was used as a housekeeping gene for normalization of the results

The expression of *Bcl-2* in lipase treated HepG-2 was determined by QRT-PCR using *Bcl-2* specific primers and the obtained results showed that *Bcl-2* gene was significantly (*P* < 0.05) downregulated by 62% following treatment of HepG-2 cells with lipase enzyme as presented in Fig. 13. In Zhao et al. (2015), *Bcl-2* was downregulated by 50% in HepG-2 cells via RNA interference, enhancing apoptosis and increasing sensitivity to chemotherapy. *Bcl-2*, an anti-apoptotic protein, helps liver cancer cells survive by preventing apoptosis [44]. This downregulation in the expression of *Bcl-2* gene would activate the production of cytochrome c which plays an important role in activating apoptotic protease-activating factor-1 (APAF1) causing the secretion of caspase-3 leading to necrosis of Hepg-2 cell line [45]. Accordingly, downregulation of *Bcl-2* suggests that lipase may more effectively induce apoptosis in liver cancer cells. This highlights lipase as a potential therapeutic alternative for targeting *Bcl-2* and promoting cell death in liver cancer [46] (Figs. 12, 13).

## Conclusion

Our results indicated that from the economic point of view, *P. aeruginosa* sp. possesses a very high capacity for extracellular lipase production. In this study, swabs from biological samples were isolated and identified by different microbiological experiments then it was subjected to a production process by submerged fermentation at a small scale. Production parameters were optimized statistically by expert design and RSM CCD, and then, it was found that the best carbon source was tween 80 (2.5 g/l), the best nitrogen source was peptone (2.5 g/l), the best pH was 9, the best olive oil concentration was 1 ml/l, the best temperature was 20 °C and best incubation time was 24 h, mutation optimizes production by two-folds, the multifactorial design optimize production by 100 units than the mutant, then enzyme was partially purified and characterized and molecular weight was determined by SDS–PAGE electrophoresis and it was close to 53 kDa. Our work demonstrated that lipase might be an ideal anticancer candidate, specific to cancer cells, for treating hepatocellular carcinoma (HepG-2) by its necrotic and apoptotic activities in addition to its effect in inducing downregulation (62%) of *Bcl-2* gene.

## Data Availability

Data is provided within the manuscript or supplementary information files.
